# Patterns of Chronic Conditions and Their Associations With Behaviors and Quality of Life, 2010

**DOI:** 10.5888/pcd12.150179

**Published:** 2015-12-17

**Authors:** John P. Barile, Sandra A. Mitchell, William W. Thompson, Matthew M. Zack, Bryce B. Reeve, David Cella, Ashley Wilder Smith

**Affiliations:** Author Affiliations: Sandra A. Mitchell, Ashley Wilder Smith, National Cancer Institute, Rockville, Maryland; William W. Thompson, Matthew M. Zack, Centers for Disease Control and Prevention, Atlanta, Georgia; Bryce B. Reeve, University of North Carolina at Chapel Hill, Chapel Hill, North Carolina; David Cella, Northwestern University, Chicago, Illinois.

## Abstract

**Introduction:**

Co-occurring chronic health conditions elevate the risk of poor health outcomes such as death and disability, are associated with poor quality of life, and magnify the complexities of self-management, care coordination, and treatment planning. This study assessed patterns of both singular and multiple chronic conditions, behavioral risk factors, and quality of life in a population-based sample.

**Methods:**

In a national survey, adults (n = 4,184) answered questions about the presence of 27 chronic conditions. We used latent class analysis to identify patterns of chronic conditions and to explore associations of latent class membership with sociodemographic characteristics, behavioral risk factors, and health.

**Results:**

Latent class analyses indicated 4 morbidity profiles: a healthy class (class 1), a class with predominantly physical health conditions (class 2), a class with predominantly mental health conditions (class 3), and a class with both physical and mental health conditions (class 4). Class 4 respondents reported significantly worse physical health and well-being and more days of activity limitation than those in the other latent classes. Class 4 respondents were also more likely to be obese and sedentary, and those with predominantly mental health conditions were most likely to be current smokers.

**Conclusions:**

Subgroups with distinct patterns of chronic conditions can provide direction for screening and surveillance, guideline development, and the delivery of complex care services.

## Introduction

The rising prevalence of chronic diseases creates a burden for individuals and society. Multiple chronic conditions (MCCs) increase the risks of death, disability, adverse events, symptom burden, impaired functional status, and lower health-related quality of life (HRQOL) (1,2). Furthermore, 66% of all health care spending is associated with providing care for the 27% of Americans with MCCs (3).

National health agencies have called for strategies to maximize care coordination and to mitigate adverse health outcomes by strengthening self-management among those with MCCs (4) and by encouraging research to inform evidence-based practice and system redesign to improve outcomes (4,5). These calls extend to strengthening mental health care systems (6) and improving our understanding of health disparities among individuals with MCCs, including those with mental illnesses (7).

Traditionally, MCCs have been assessed through broad summary scores. However, because these scores do not reflect the patterns of chronic conditions, substantial gaps remain in understanding interactions among chronic conditions and the effect these interactions have on treatment. These knowledge gaps limit our ability to deliver effective and efficient health care in the predominantly single-disease–focused care delivery model.

Chronic conditions can act cumulatively and synergistically to adversely affect health outcomes, caregiver burden, and treatment costs (8–10). For several diseases, adding just 1 additional disease to the index disease markedly worsens HRQOL (10,11); functional limitations partially account for these adverse effects (12). Knowledge of patterns of chronic conditions, their effects on HRQOL, and their association with health behaviors could inform interventions to prevent or preempt MCCs, reduce their burden (13,14), and optimize service delivery (15,16) for individuals with chronic conditions (13,17).

The objectives of this study were to use latent class analysis (LCA) to examine 1) co-occurrence patterns of 27 self-reported chronic health conditions in a large, nationally representative adult sample, and 2) whether these patterns were associated with sociodemographic factors, tobacco use, physical activity, body mass index (BMI), and differences in self-assessed health status and well-being.

## Methods

### Sample and setting

The data were obtained from the summer wave of Porter Novelli’s 2010 HealthStyles database. Each year, the HealthStyles database is collected from several mailed panel surveys that gather information on the health of US adults. The sampling design included stratification by region, yearly household income, population density, age, and household size. In August and September of 2010, of 6,255 adults aged 18 years or older, 4,184 (66.9%) responded to these mailed surveys. Respondents received $5 and entry into a lottery (1 first-place prize of $1,000 and 20 second-place prizes of $50) as compensation for their time. Analyzed data excluded personal identifiers.

Survey data were demographically weighted to match US population estimates. Of the sample respondents, 49% were male, 69% were white, 12% were black, and 14% were Hispanic/Latino. They included adults aged 18 to 24 (13%), 25 to 34 (18%), 35 to 44 (18%), 45 to 54 (20%), 55 to 64 (15%), and 65 or older (17%). Twenty-five percent had a yearly household income less than $25,000 and 43% had a yearly household income of $60,000 or more. Fifty-five percent were married, and 32% were college graduates.

### Measures

Respondents answered the following question with respect to 34 medical conditions: “During the past year, have you had (or do you currently have) any of these health conditions?” For our analyses, we excluded seasonal allergies and influenza because they are not considered to be a chronic condition. We also excluded male erectile dysfunction and enlargement of the prostate because they affect only men. Because of low prevalence, we combined the 4 cancer types — prostate, breast, lung, and other cancer — into a single cancer variable, “Cancer other than skin cancer.” Table 1 lists the 27 chronic conditions studied and their weighted sample prevalences.

**Table 1:** Frequencies of the Chronic Conditions Included in the Latent Class Analyses of Associations With Behaviors and Quality of Life (n = 4,184), HealthStyles Survey, 2010^a^

Condition	n^b^ (Weighted %)
Multiple sclerosis	39 (1)
Epilepsy or seizure disorder	52 (1)
Congestive heart failure	70 (1)
Atrial fibrillation	98 (3)
Ulcers	105 (3)
Other mental health condition	116 (3)
Emphysema or chronic obstructive pulmonary disease	131 (3)
Other heart disease	155 (3)
Skin cancer	156 (3)
Heart disease (angina or myocardial infarction)	158 (3)
Prostate, breast, lung, or other cancer	175 (3)
Eczema	180 (6)
Osteoporosis	203 (5)
Irritable bowel syndrome	214 (5)
Sciatica	222 (5)
Overactive bladder or incontinence	329 (7)
Asthma	386 (10)
Hearing impairment	393 (8)
Insomnia or sleep disorder	464 (12)
Migraine headaches	532 (14)
Anxiety	593 (16)
Depression	631 (16)
Diabetes	645 (14)
Chronic pain	659 (15)
High cholesterol	1,148 (25)
Arthritis	1,153 (24)
High blood pressure	1,468 (31)

Source: Porter Novelli’s 2010 HealthStyles database.

a Of the sample, 26.2% reported no chronic conditions, 20.8% reported 1 condition, and 53.0% reported 2 or more conditions.

b Unweighted sample size.

Respondents were asked whether they had ever smoked at least 100 cigarettes in their life. If so, they were asked if they currently smoked every day, some days, or not at all. We considered respondents who had smoked 100 cigarettes but did not currently smoke to be former smokers.

We estimated physical activity levels from the number of days per week in a “usual week” and the number of minutes per day that participants reported engaging in either vigorous or moderate physical activity. We also used the number of days per week they performed muscle-strengthening activities. We categorized responses based on federal guidelines (18).

We calculated BMI from the respondents’ self-reported weight and height (BMI = weight [lbs] ÷ height [in]^2^ × 703). We then categorized respondents as underweight, normal weight, overweight, or obese using World Health Organization thresholds (19). 

The HealthStyles Survey used the National Institutes of Health Patient-Reported Outcomes Measurement Information System (PROMIS) Global Health Scale, the Centers for Disease Control and Prevention (CDC) Healthy Days measures, and 4 items from the Satisfaction With Life Scale (SWLS) (20). The PROMIS scale has 10 items that assess physical, mental, and social aspects of health (14,15). The 4 core CDC Healthy Days measures (http://www.cdc.gov/hrqol/methods.htm) on the HealthStyles survey assess general self-rated health, physically unhealthy days, mentally unhealthy days, and activity limitation days (16). The Appendix provides additional information about these measures.

### Analytic procedures

We conducted LCA using Mplus 7.3 (http://statmodel.com) to identify classes or subgroups of respondents with distinct patterns of co-occurring chronic conditions. LCA is a person-centered statistical approach for identifying subgroups of people who share similar characteristics (21). In this analysis, the presence or absence of 27 chronic health conditions was used to divide respondents into latent subgroups that share a distinct interpretable pattern of relationships among the conditions. LCA assumes that at least 2 subgroups can be identified within a sample and that a categorical latent variable indicating membership in a subgroup explains the relationship among the observed conditions. LCA therefore permits subgroups of respondents with differing profiles of co-occurring chronic conditions to be determined probabilistically based on their responses to questions about the presence of 27 health conditions.

We assessed a series of LCA models beginning with a single-class model and adding classes until model fit no longer significantly improved. We chose a best-fitting model based on the Akaike information criterion (AIC) and the Bayesian information criterion (BIC) (22). We did not use other common criteria, such as the bootstrap likelihood ratio test, because the survey analysis required accounting for respondent sampling weights. We also inspected probability plots for the latent classes to consider the substantive interpretability (meaningfulness and distinctiveness) of the resultant latent class solutions.

After estimating the number of latent classes, age, sex, yearly household income, and race/ethnicity were used as covariates to assess improvement in model fit. We then assessed the relationships between latent class membership and the behavioral risk factors tobacco use, exercise, and BMI. Last, we examined the relationships between latent class membership and scores on the PROMIS global physical and mental health scales and the CDC Healthy Days measures to determine the predictive validity and clinical utility of the latent classes (23).

## Results

LCA models estimating 2, 3, and 4 latent classes converged normally. Of the 3 models, the 4-class solution resulted in the best fit (111 parameters; AIC = 53,650; BIC = 54,354; sample-size adjusted BIC = 54,001; and a comparable entropy value = 0.76). The 2-class solution resulted in a worse fit (55 parameters; AIC = 55,263; BIC = 55,612; sample-size adjusted BIC = 55,437; entropy = 0.78) as did the 3-class solution (83 parameters; AIC = 54,127; BIC = 54,653 sample-size adjusted BIC = 54,390; entropy = 0.78).

We also examined the average posterior probabilities (where values closer to 1 are interpreted as reflecting better class separation) across the 3 solutions. The posterior probabilities ranged from 0.90 to 0.95 for the 2-class solution, from 0.86 to 0.91 for the 3-class solution, and from 0.82 to 0.89 for the 4-class solution. The 4-class model was selected as the best model because it resulted in good model fit, maintained adequate class separation, and revealed interpretable subgroups.

The fit of the 4-class model further improved with the inclusion of the covariates (AIC = 53,650 without covariates, AIC = 52,375 with covariates; BIC = 54,354 without covariates, BIC = 53,193 with covariates). All results presented hereafter reflect the inclusion of these covariates.

The class-specific probabilities of reporting each of the 27 chronic conditions for the 4-class solution appear in the Figure. On the basis of these distinct patterns of chronic conditions, we named class 1 the healthy class (HC) because this subgroup had the lowest probability of reporting any of the conditions. HC was represented by 54.1% of the sample and reported an average of 0.80 conditions (standard deviation [SD] = .92). Class 2 (the physical health conditions class [PHCC]) represents people who have a high probability of reporting high blood pressure (58%), high cholesterol (45%), and diabetes (26%) as well as conditions closely associated with aging, such as cancer (6%) and hearing impairment (16%). PHCC was represented by 27% of the sample and across all of the conditions and reported an average of 3.03 conditions (SD = 1.42). We named class 3 the mental health conditions class (MHCC), because people in this subgroup had high probabilities of reporting mental health conditions (72% reported depression, 70% reported anxiety) but low probabilities of reporting physical health conditions except for migraine headaches, chronic pain, sciatica, asthma, eczema, and insomnia. MHCC was represented by 10.5% of the sample and across all of the conditions and reported an average of 5.04 conditions (SD = 2.15). We named class 4 the physical and mental health conditions class (PMHCC) because it included people who were highly likely to report multiple physical health conditions, particularly arthritis (83%) and osteoporosis (26%) as well as mental health conditions (49% reported depression and 49% reported anxiety). PMHCC was represented by 8.3% of the sample and across all of the conditions and reported an average of 7.77 conditions (SD = 2.94). Finally, these classes differed significantly based on sociodemographic characteristics. The HC had significantly higher income than the other classes, the PHCC was much older and had more African Americans/blacks than the other classes, and the MHCC had more women and younger persons. The Appendix more completely describes these associations between the latent classes and sociodemographic characteristics.

**Figure:**
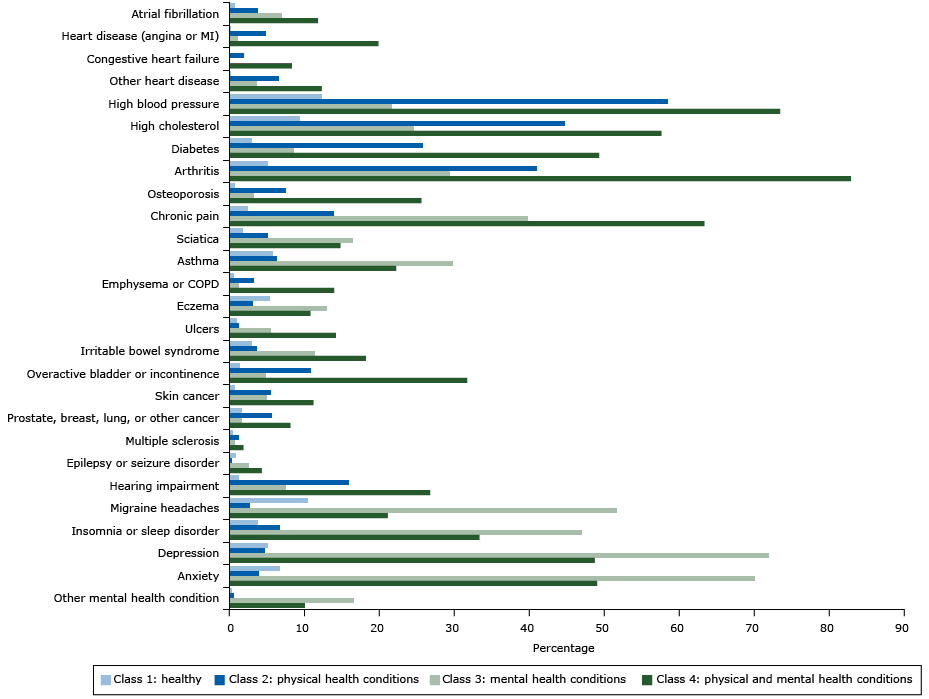
Estimated probabilities of reporting diseases or conditions, by class (not adjusted for overall prevalence), in analysis of associations of behaviors and quality of life (n = 4,184), HealthStyles Survey, 2010. All probabilities were adjusted for age, race/ethnicity, sex, and yearly household income. Abbreviations: COPD, chronic obstructive pulmonary disease; MI, myocardial infarction. **Table:** 

Chronic Condition	Class 1: Healthy	Class 2: Physical Health Conditions	Class 3: Mental Health Conditions	Class 4: Physical and Mental Health Conditions
Atrial fibrillation	0.01	0.04	0.07	0.12
Heart disease (angina or MI)	0	0.05	0.01	0.20
Congestive heart failure	0	0.02	0	0.08
Other heart disease	0	0.06	0.04	0.12
High blood pressure	0.12	0.58	0.22	0.73
High cholesterol	0.09	0.45	0.25	0.58
Diabetes	0.03	0.26	0.08	0.49
Arthritis	0.05	0.41	0.29	0.83
Osteoporosis	0.01	0.07	0.03	0.26
Chronic pain	0.02	0.14	0.40	0.63
Sciatica	0.02	0.05	0.16	0.15
Asthma	0.06	0.06	0.30	0.22
Emphysema/COPD	0.01	0.03	0.01	0.14
Eczema	0.05	0.03	0.13	0.11
Ulcers	0.01	0.01	0.05	0.14
Irritable bowel syndrome	0.03	0.04	0.11	0.18
Overactive bladder or incontinence	0.01	0.11	0.05	0.32
Skin cancer	0.01	0.06	0.05	0.11
Prostate, breast, lung, other cancer	0.02	0.06	0.02	0.08
Multiple sclerosis	0	0.01	0.01	0.02
Epilepsy or seizure disorder	0.01	0	0.03	0.04
Hearing impairment	0.01	0.16	0.07	0.27
Migraine headaches	0.10	0.03	0.52	0.21
Insomnia or sleep disorder	0.04	0.07	0.47	0.33
Depression	0.05	0.05	0.72	0.49
Anxiety	0.07	0.04	0.70	0.49
Other mental health condition	0	0.01	0.16	0.10

### Association of the latent classes with HRQOL, well-being, and behavioral risk factors

The 4 latent classes also differed by tobacco use, adherence to exercise guidelines, and BMI (Table 2). Those in the MHCC were most likely to currently smoke compared with all other classes, those in the PHCC were the least likely to currently smoke (although 38% were former smokers). Compared with the other groups, those in the PMHCC reported being more sedentary, were least likely to meet minimal or maximal aerobic guidelines or to perform strength training activities, and were more likely to be obese or overweight.

**Table 2:** Frequency of Behavioral Risk Factors, by Latent Class Membership in Analyses of Associations With Behaviors and Quality of Life (n = 4,184), HealthStyles Survey, 2010^a^

Behavior	Total, %	Class 1: Healthy, %	Class 2: Physical Health Conditions, %	Class 3: Mental Health Conditions, %	Class 4: Physical and Mental Health Conditions, %
**Tobacco use**					
Nonsmoker	57.5	65.9	48.1	52.4	36.7
Former smoker	24.8	18.0	38.0	13.7	41.7
Current smoker	17.7	16.1	13.9	33.8	21.6
**Body mass index (BMI), kg/m^2^**					
Underweight, BMI <18.5	1.6	1.9	1.5	0.9	0.8
Normal weight, BMI 18.5–24.9	29.0	34.7	24.2	28.6	14.1
Overweight, BMI 25.0–29.9	32.4	33.4	34.1	30.9	31.1
Obese, BMI ≥30.0	34.6	29.9	40.2	39.7	53.9
**Leisure-time aerobic activity, min/wk**					
Sedentary (0)	16.8	12.8	21.5	15.7	31.4
Low aerobic, below guidelines (1–149)	26.8	24.2	30.5	26.8	33.1
Meets minimal aerobic guidelines (150–299)	19.9	21.6	18.1	19.7	14.0
Meets maximal aerobic guidelines (≥300)	36.5	41.4	29.9	37.8	21.5
**Strength training^b^**					
Low or none (0 or 1 time per week)	57.1	54.7	61.1	53.2	67.1
Moderate (≥2 times per week)	42.9	43.2	38.9	46.8	32.9
**Aerobic activity and strength training^b^**					
Sedentary (0 min/wk)	16.0	12.1	20.7	14.9	30.5
Meets recommended strength training only^b^	27.0	24.5	30.3	27.0	34.3
Low aerobic only	10.2	10.6	10.2	10.9	6.8
Meets minimal or maximal aerobic guidelines without recommended strength training	24.0	27.1	20.5	20.0	18.6
Meets minimal or maximal aerobic guidelines with recommended strength training	22.7	25.7	18.3	27.2	9.7

a All percentages correspond to the weighted sample.

b Federal guidelines recommend muscle-strengthening activities on 2 or more days per week.

People in the HC reported the fewest physically unhealthy days, the fewest days with activity limitations, and higher PROMIS Physical health T-scores (all *P* < .001; Table 3). Wellbeing, mentally unhealthy days, and PROMIS mental health T-scores in the HC and PHCC were comparable and significantly better than the well-being, mentally healthy days, and PROMIS mental health T-scores reported by members of the other 2 classes. Those in the PMHCC reported significantly more physically unhealthy and activity limited days as well as lower physical health T-scores (all *P* < .001) and lower well-being scores (*P* < .05) than all other classes.

**Table 3:** Mean Differences on Measures of Health-Related Quality of Life and Well-Being by Latent Class in Analyses of Associations With Behaviors and Quality of Life (n = 4,184), HealthStyles Survey, 2010^a^

Measure	Class 1: Healthy, Mean^b^ (95% CI)	Class 2: Physical Health Conditions, Mean^b^ (95% CI)	Class 3: Mental Health Conditions, Mean^b^(95% CI)	Class 4: Physical and Mental Health Conditions, Mean^b^ (95% CI)
Physically unhealthy days (range, 0–30)^c^	0.9 (0.7–1.1)	4.8 (4.1–5.5)	5.8 (5.4–6.3)	23.4 (22.7–24.0)
Mentally unhealthy days (range, 0–30)^c^	1.4 (1.2–1.6)	0.8 (0.2–1.5)	6.2 (5.8–6.7)	5.2 (4.3–6.0)
Activity limitation days (range, 0–30)^c^	0.4 (0.2–0.6)	3.8 (3.2–4.4)	5.2 (4.7–5.6)	21.1 (20.5–21.7)
PROMIS physical health T-score^d^	52.3 (52.1–52.6)	47.0 (46.3–47.8)	43.2 (42.7–43.8)	38.4 (37.7–39.2)
PROMIS mental health T-score^d^	50.0 (49.7–50.2)	49.4 (48.7–50.2)	43.7 (43.2–44.3)	43.5 (42.7–44.2)
Well-being score (range, 1–5)^e^	3.6 (3.6–3.7)	3.6 (3.5–3.7)	3.0 (2.9–3.1)	2.6 (2.5–2.7)

Abbreviations: CI, confidence interval; PROMIS, Patient-Reported Outcomes Measurement Information System.

a Overall means: physically unhealthy days = 3.24 (standard deviation [SD] = 7.49); mentally unhealthy days = 2.51 (SD = 6.23); activity limitation days = 2.22 (SD = 6.29); PROMIS physical health = 49.86 (SD = 8.29); PROMIS mental health = 49.47 (SD = 7.89); well-being score = 3.46 (SD = 0.91). All means correspond to the weighted sample.

b Estimated marginal means controlled for age, race/ethnicity, sex, and yearly household income. All means correspond to the weighted sample.

c Measured by using the Centers for Disease Control and Prevention’s Healthy Days measures (http://www.cdc.gov/hrqol/methods.htm).

d Measured by using the National Institutes of Health PROMIS Global Health Scale (14,15).

e Measured by using the Satisfaction with Life Scale (20).

## Discussion

This study provides new evidence about the patterns of chronic conditions and their associations with behavioral risk factors, HRQOL, and well-being. Using a nationally representative sample and applying latent class analysis to 27 chronic health conditions, we identified 4 distinct profiles of chronic conditions. Although extensive evidence associates overweight, obesity, sedentary lifestyle, and tobacco use with chronic diseases (24), this study empirically derives patterns of co-occurring conditions and examines the association of those patterns with HRQOL and behavioral risk factors, thereby generating population-level insights that may have implications for health care delivery.

The 4 profiles of chronic conditions and their associations with behavioral risk factors and health outcomes suggest the need to treat common patterns of chronic conditions simultaneously, thereby improving efficiency and cost-effectiveness. For example, in tailoring interventions for the PHCC (people who very probably reported co-occurring physical conditions such as hypertension, high cholesterol, diabetes, and obesity but who maintained good health status and well-being and no activity limitations), interventions should focus on the continuity of care for hypertension, dyslipidemia, and diabetes and on physical activity interventions emphasizing aerobic exercise and strength training. This package of interventions could simultaneously improve disease management outcomes, decrease BMI, reduce risk factors for cardiovascular disease, and prevent the onset of disability. In contrast, those in the PMHCC report various serious physical conditions, anxiety, and depression. They also reported more activity limitations, more unhealthy days, and poorer well-being. This group may warrant a well-coordinated, interdisciplinary care delivery approach that emphasizes physical rehabilitation and exercise, mental health screening, and self-management interventions. For the class with mostly mental health conditions who tended to be younger and female, targeting delivery of mental health services to a younger population (eg, addressing stigma) and management of migraine headache, chronic pain, and insomnia may improve outcomes. This group may warrant efforts to reduce smoking and to improve exercise, and energy balance, since 33% currently smoked, 71% were overweight or obese, and about 25% met daily recommended requirements for exercise.

Our results also imply that screening and surveillance should be tailored and guidelines developed for persons with MCCs. Our observation that people with multiple physical conditions including atrial fibrillation, heart disease, osteoporosis, and cancer are also likely to have anxiety or depression or both suggests the importance of regular periodic screening for these mental disorders and the potential value of incorporating mental health interventions into disease management algorithms for these specific conditions (25). At the same time, guidelines for the treatment of mental health conditions should address the need for primary prevention strategies, including smoking cessation and diet and physical activity interventions.

We were intrigued that respondents in the group with hypertension, dyslipidemia, and diabetes (PHCC) reported only modestly lower PROMIS physical health T-scores compared with the HC, despite reporting 4.8 physically unhealthy days and 3.8 days of activity limitations. These observations suggest that physically unhealthy days and activity limitation days may provide a responsive initial signal of preclinical disability, particularly in older adults with hypertension, dyslipidemia, or cancer and who are sedentary or obese or both. For clinicians in primary care, these brief measures may help identify patients with MCCs who also have functional health impairments. Prospective observational studies are needed to determine the sensitivity and responsiveness of these brief indicators for earliest detection of subtle adverse changes in health status in people with MCCs.

Only 16% of the HC while 34% of the MHCC reported that they were current smokers. A large proportion of the PHCC and the PMHCC were former smokers. Worse mental health is associated with tobacco use, and the results of this study support the need for tobacco use cessation interventions and other public health strategies focused specifically on people with mental illnesses (26). The former smokers in the PHCC and the PMHCC may have stopped smoking for different reasons, including the occurrence of chronic conditions associated with their previous tobacco use. Longitudinal studies are needed to determine how membership in 1 of the 4 subgroups transitions across time, particularly as a function of continued tobacco use, and whether people in certain classes are at greater risk for the adverse health effects of tobacco use. For example, those in the MHCC who continue to smoke may ultimately transition into the subgroup with both physical and mental conditions (27). These findings also highlight the need for longitudinal studies to determine whether particular profiles of co-occurring chronic conditions amplify the risks for smoking-related health impairments.

Respondents with co-occurring physical and mental health conditions were also most likely to be obese and to fail to meet guidelines for aerobic exercise and strength training. This observation, together with the prominent activity limitations and impaired HRQOL also observed in this subgroup, suggest particular clinical challenges when implementing traditional lifestyle management interventions. Although aerobic exercise and strength training favorably affect chronic disease outcomes, the best approaches to tailoring diet and exercise interventions for people with MCCs and monitoring progress toward goals deserve further study (28,29).

Some caveats are necessary when interpreting our findings. The survey was a mailed survey with only a 66.9% response rate. Although this response rate is comparable to other nationally representative surveys, the mode of administration and the response rate could indicate a nonrepresentative sample. Because the chronic conditions were self-reported, some misclassification of chronic conditions may have occurred (eg, misreporting conditions such as arthritis, arthrosis, and osteoporosis), and the prevalence rates for some conditions were low. Collapsing conditions (eg, cancer other than skin cancer) into a single category may have limited our ability to distinguish differences in the co-occurrence patterns. Additionally, the survey asked about only 2 specific mental health conditions (depression and anxiety) and these findings could shift with inclusion of other mental health conditions.

Future research should examine whether our findings could be replicated using other nationally representative samples, including those with older adults. Furthermore, longitudinal data would allow for the examination of whether people transition from one class to another over time, for example, from the MHCC to the PMHCC. We found that the total number of chronic conditions differed between classes (79% of HC report 0 or 1 condition while the 100% of the PMHCC reported 4 or more conditions). This finding suggests that individuals may transition from one class to another as they develop additional conditions. In particular, an investigation into potential transitions across classes should be examined in light of known associations between conditions (30)

US and international health agencies continue to devise and revise national strategies to maximize coordination of care, reduce health burden, and improve HRQOL. Using LCA indicated a taxonomy of chronic conditions that, if replicated, could prove useful for planning, delivering, and evaluating disease management programs and strategies to improve population health.

## Appendix

This file is available for download as a Microsoft Word document.
