# Supplementary material for: Patterns of Chronic Conditions and Their Associations With Behaviors and Quality of Life, 2010
Source: Prev Chronic Dis. 2015 Dec 17;12:E222. doi: 10.5888/pcd12.150179 (PMC5241639; doi:10.5888/pcd12.150179)
Supplement: Supplementary file 1 [file 15_0179_Appendix.docx]

**APPENDIX**

**Additional Information on Measures**

The National Institutes of Health Patient-Reported Outcomes Measurement Information System (PROMIS) scale includes 10 items that tap into physical, mental, and social aspects of health. In a previous psychometric study (1), PROMIS investigators identified 2 overall factors — physical health and mental health. However, because 1 item (“In general, please rate how well you carry out your usual social activities and roles.”) did not load on either factor, PROMIS does not use it to estimate their general population physical and mental health summary T-scores. The creation of PROMIS T-scores was based on prior work by Hays and colleagues (1) and Cella and colleagues (2). PROMIS global physical health and mental health T-scores have a mean of 50 and a standard deviation of 10, with higher scores indicating better health.

The Centers for Disease Control and Prevention (CDC) Healthy Days measures (http://www.cdc.gov/hrqol/methods.htm) has 4 core items and 10 supplemental items. The 4 core items on the HealthStyles survey measured general self-rated health, physically unhealthy days, mentally unhealthy days, and activity limitation days. The general self-rated health item (“Would you say that in general your health is – Excellent, Very Good, Good, Fair, or Poor?”) is the same item as the one included on the PROMIS Global Health Scale. The CDC items have demonstrated content, construct, and criterion validity (3-7).

Well-being was measured by 4 items from the Satisfaction With Life Scale (SWLS) (8). The SWLS has shown acceptable test–retest reliability and is sensitive to life events (9-14). The SWLS asks participants to report how much they agreed, from strongly disagree to strongly agree (a 5-point Likert-type scale), with 4 statements (“In most ways my life is close to my ideal.”; “The conditions of my life are excellent.”; “I am satisfied with my life.”; “So far I have gotten the important things I want in life.”). The fifth item from the SWLS (“If I could live my life over, I would change almost nothing.”) was excluded from the HealthStyles survey to limit respondent burden without substantially reducing the reliability or validity of the 4-item scale (15).

To calculate physical activity levels, respondents answered how often they engaged in various forms of leisure-time physical activity. Participants reported the usual number of days per week and minutes per day they engaged in either vigorous or moderate physical activity as well as the number of days per week they performed muscle-strengthening activities. Responses were then categorized based on current federal guidelines (16). These guidelines recommend that American adults engage in at least 150 minutes of moderate-intensity or 75 minutes of vigorous-intensity physical activity each week (or an equivalent combination). For more extensive health benefits, double that level is recommended. For this analysis, respondents’ weekly physical activity levels were coded as follows: sedentary = 0 min/wk; low aerobic activity/below guidelines = 1–149 min/wk; meets minimal aerobic guidelines = 150–299 mi/wk; or meets maximal aerobic guidelines = 300 or more min/wk). Federal guidelines also recommend muscle-strengthening activities on 2 or more days per week. Respondents also answered how often they engaged in exercises specifically designed to strengthen their muscles, such as lifting weights or doing calisthenics. Respondents engaging in strength training less than twice per week were recoded as not engaging in moderate strength training and those who reported doing so 2 or more times a week were considered to engage in moderate strength training.

**Latent Class Analysis Adjusted for Prevalence by Condition**

Results of the latent class analysis after adjustment for the total prevalence of each condition is depicted in Appendix Figure 1. The Y axis represents the percentage of participants that reported being diagnosed with each condition out of the total number of reports across all classes. This representation of the data improves the readability of the Figure in the main text for low prevalence conditions (1% of the total sample or less). Findings from this depiction of the data suggest that multiple sclerosis and congestive heart failure were most commonly reported by individuals in the Physical and Mental Health Conditions class and the Physical Health Conditions class. Epilepsy or seizure disorders were found to be most common among individual in the Physical and Mental Health Conditions class followed by those in the Mental Health Conditions class.

**Sociodemographic Characteristics of the 4 Comorbidity Classes**

When estimating the LCA models, we included the following demographic covariates: Yearly household income, age, sex, and race/ethnicity (Appendix Figures 2, 3, 4, and 5). Appendix Figure 2 presents the estimated probabilities that people with different reported yearly household income had of being included in each of the 4 classes. The probability of being included in the Healthy Class compared to all other classes increased as yearly household income increased (P < .05). The probability of being classified into the Physical and Mental Health Conditions Class, compared to all other classes, declined as yearly household income increased (*P* < .05). The probability of being in the Physical Health Conditions Class or the Mental Health Conditions Class remained relatively stable across all income levels.

Appendix Figure 3 presents the estimated probabilities that individuals in different age groups have of being included in each of the 4 latent classes. As age increased, individuals had a much higher probability of being included in the Physical Health Conditions Class and a decreasing probability of being included in the Healthy Class (*P* < .001). Age played less of a role in determining whether individuals would be classified in the Physical and Mental Health Conditions Class and the Mental Health Conditions Class.

Appendix Figure 4 presents the estimated probabilities of being placed in each of the latent classes based on sex. Sex played a notable role only in the probability of being in the Physical Health Conditions Class, with men being more likely to be included, and in the Mental Health Conditions Class, with women being more likely to be included (*P* < .05).

Appendix Figure 5 presents the estimated probabilities of being placed in each of the latent classes based on race/ethnicity. Compared with adults in other ethnic groups in the other classes, black adults had a higher probability of being included in the Physical Health Conditions Class than other races/ethnicities but a lower probability of being included in the Physical and Mental Health Conditions Class (*P* < .05) and the Mental Health Conditions Class (*P* < .01). No other race/ethnicity effects were significant.

**Appendix References**

1. Hays RD, Bjorner JB, Revicki DA, Spritzer KL, Cella D. Development of physical and mental health summary scores from the Patient-Reported Outcomes Measurement Information System (PROMIS) global items. Qual Life Res 2009;18(7):873–80.

2. Cella D, Riley W, Stone A, Rothrock N, Reeve B, Yount S, et al. The Patient-Reported Outcomes Measurement Information System (PROMIS) developed and tested its first wave of adult self-reported health outcome item banks: 2005–2008. J Clin Epidemiol 2010;63(11):1179–94.

3. Centers for Disease Control and Prevention. Measuring Healthy Days. Atlanta (GA): Centers for Disease Control and Prevention; 2000.

4. Jiang Y, Hesser JE. Using item response theory to analyze the relationship between health-related quality of life and health risk factors. Prev Chronic Dis 2009;6(1):A30.

5. Mielenz T, Jackson E, Currey S, DeVellis R, Callahan LF. Psychometric properties of the Centers for Disease Control and Prevention Health-Related Quality of Life (CDC HRQOL) items in adults with arthritis. Health Qual Life Outcomes 2006;4:66–84.

6. Moriarty DG, Kobau R, Zack MM, Zahran HS. Tracking Healthy Days — a window on the health of older adults. Prev Chronic Dis 2005;2(3):A16.

7. Moriarty DG, Zack MM, Kobau R. The Centers for Disease Control and Prevention’s Healthy Days Measures — population tracking of perceived physical and mental health over time. Health Qual Life Outcomes 2003;1:37.

8. Diener E, Emmons RA, Larsen RJ, Griffin S. The Satisfaction With Life Scale. J Pers Assess 1985;49:71–5.

9. Blais MR, Vallerand RJ, Pelletier LG, Brière NM. L’échelle de satisfaction de vie: Validation canadienne-française du “Satisfaction with Life Scale.” Can J Behav Sci 1989;21(2):210–23.

10. Larsen RJ, Eid M. Ed Diener and the science of subjective well-being. In: Eid M, Larsen RJ, editors. The science of subjective well-being. New York (NY): Guilford Publications, Inc; 2008:1–13.

11. Magnus K, Diener E, Fujita F, Pavot W. Extraversion and neuroticism as predictors of objective life events: a longitudinal analysis. J Pers Social Psychol 1993;65(5):1046–53.

12. Pavot W. The assessment of subjective well-being. In: Eid M, Larsen RJ, editors. The science of subjective well-being. New York (NY): Guilford Publications, Inc; 2008:124–140.

13. Pavot W, Diener E. The Satisfaction With Life Scale and the emerging construct of life satisfaction. J Positive Psychol 2008;3(2):137–52.

14. Pavot W, Diener E, Colvin CR, Sandvik E. Further validation of the Satisfaction With Life Scale: evidence for the cross-method convergence of well-being measures. J Pers Assess 1991;57:149–61.

15. Kobau R, Sniezek J, Zack MM, Lucas RE, Burns A. Well-being assessment: an evaluation of well-being scales for public health and population estimates of well-being among US adults. Appl Psychol: Health Well Being 2010;2:272–97.

16. US Department of Health Human Services. 2008 Physical activity guidelines for Americans. http://www.health.gov/paguidelines/pdf/paguide.pdf. Accessed February 8, 2015.

Appendix Figure 1. The proportion of respondents that reported a history of each condition by class (ie, the percentage of respondents that accounted for the total number of reported cases for each condition) in analysis of chronic conditions and their associations with behaviors and quality of life, 2010. All probabilities were adjusted for age, race/ethnicity, sex, and yearly household income. Abbreviations: Abbreviations: MI, myocardial infarction; COPD, chronic obstructive pulmonary disease.


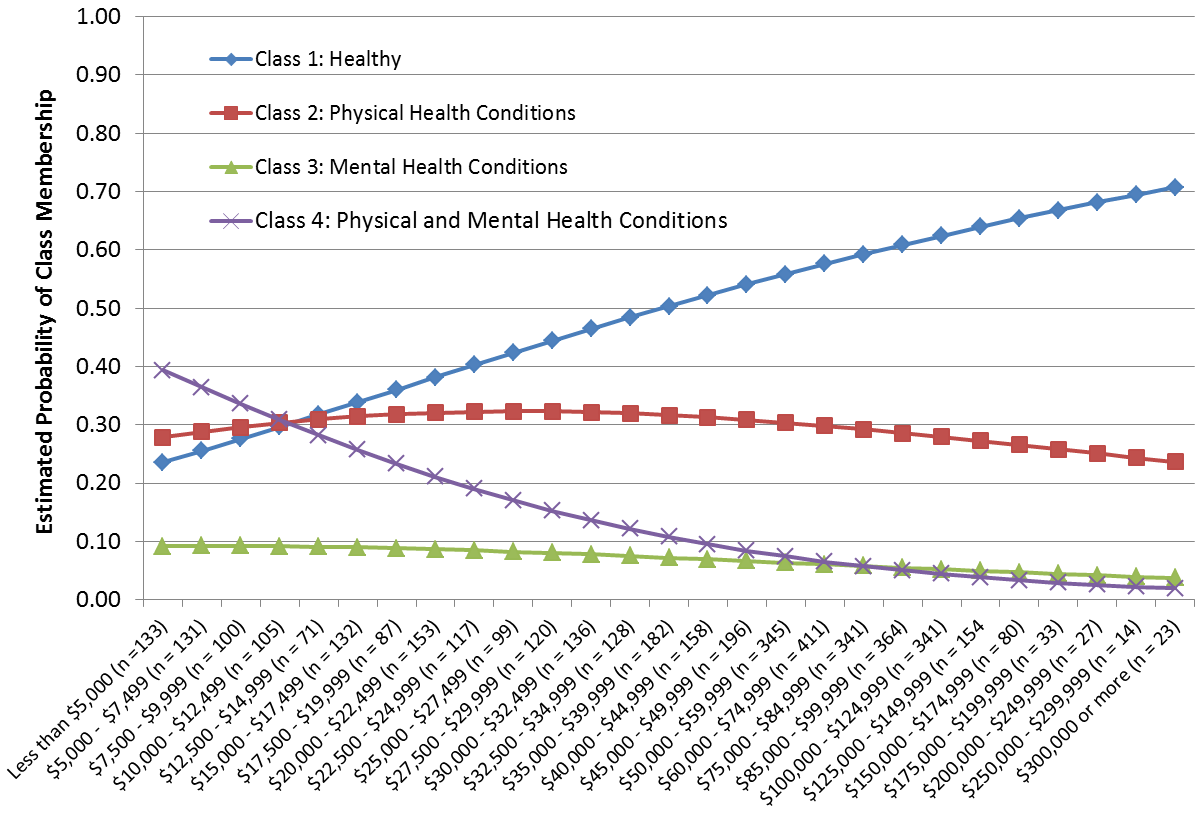
Appendix Figure 2. Estimated probability of class membership as a function of yearly household income while adjusting for age, race, and sex in analysis of chronic conditions and their associations with behaviors and quality of life, 2010.


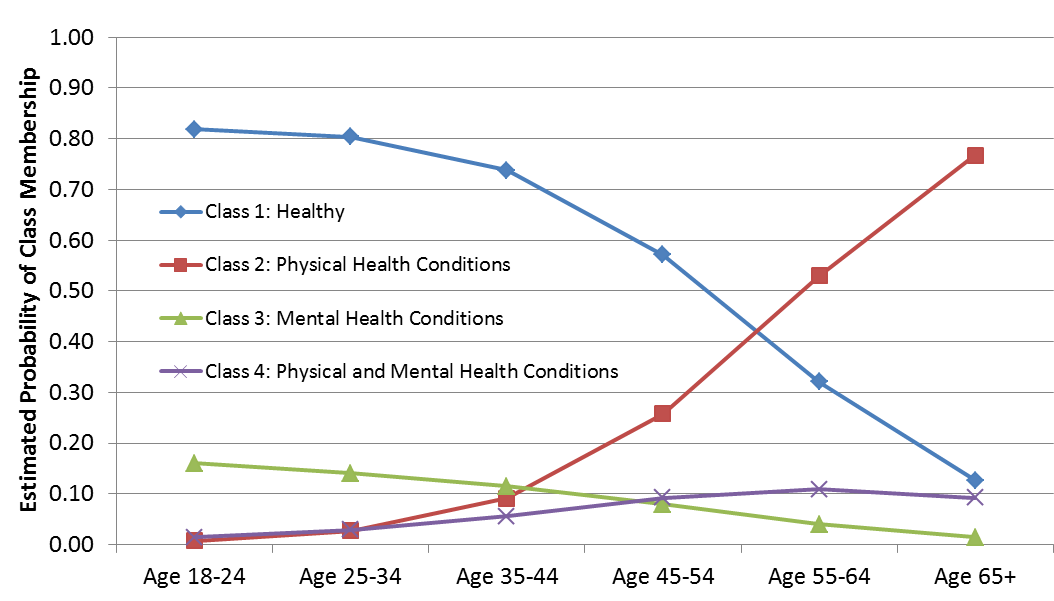


Appendix Figure 3. Estimated probability of class membership as a function of age while adjusting for yearly household income, race, and sex in analysis of chronic conditions and their associations with behaviors and quality of life, 2010.


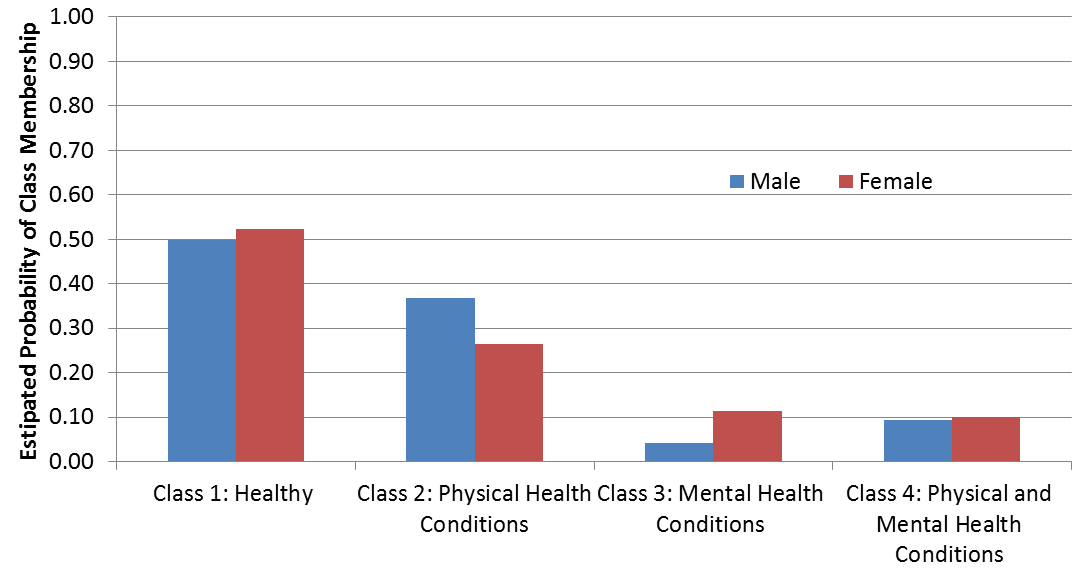
Appendix Figure 4. Estimated probability of class membership as a function of sex while adjusting for age, race, and yearly household income in analysis of chronic conditions and their associations with behaviors and quality of life, 2010.


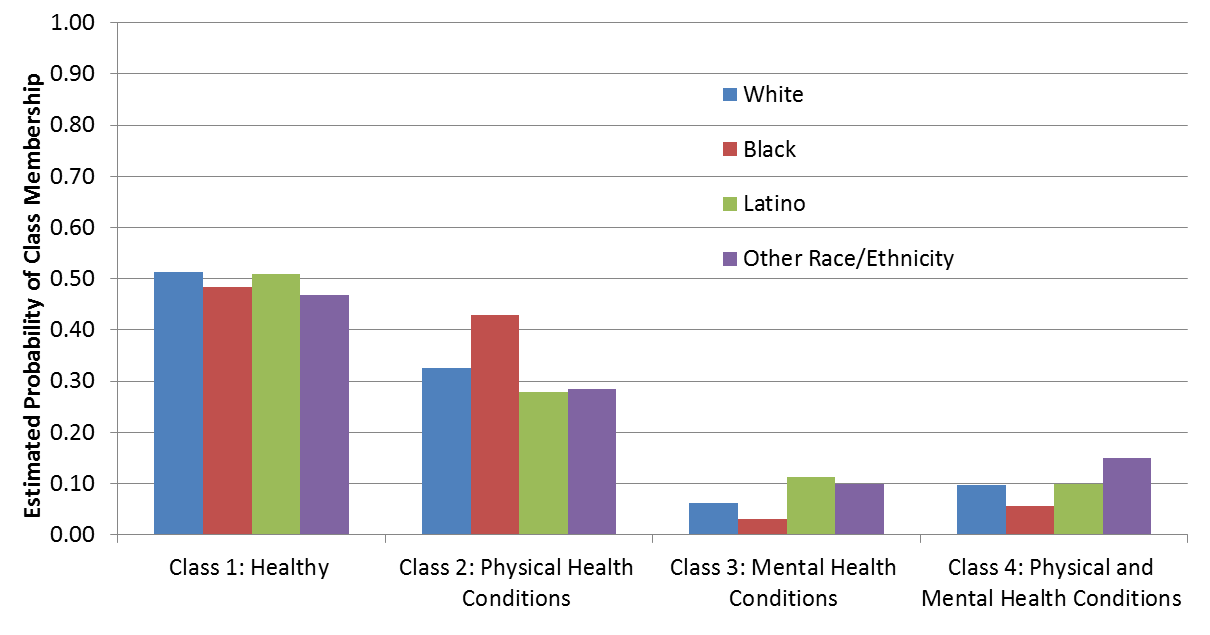
Appendix Figure 5. Estimated probability of class membership as a function of race/ethnicity while adjusting for age, yearly household income, and sex in analysis of chronic conditions and their associations with behaviors and quality of life, 2010.
